# Enhanced classification of medicinal plants using deep learning and optimized CNN architectures

**DOI:** 10.1016/j.heliyon.2025.e42385

**Published:** 2025-01-30

**Authors:** Hicham Bouakkaz, Mustapha Bouakkaz, Chaker Abdelaziz Kerrache, Sahraoui Dhelim

**Affiliations:** a Fondamental Sciences Laboratory, Université Amar Telidji de Laghouat, Laghouat, Algeria; b Laboratoire d'Informatique et de Mathématiques, Université Amar Telidji de Laghouat, Laghouat, Algeria; c School of Computing, Dublin City University, Dublin, Ireland

**Keywords:** Medicinal plant, Classification, CNN, Chimp optimization, Feature fusion

## Abstract

This work highlights the medicinal flora, which is very essential for the conservation of biodiversity and the improvement of health throughout the world. More specifically, it underlines the need for accurate classification of medicinal plant species for their effective conservation and proper use. The complexity of plant features and a lack of annotated datasets make them difficult for traditional classification methods. To address this issue, a deep learning-based framework is proposed in the research for classifying images related to medicinal plants using convolutional neural networks (CNNs). In this framework, a CNN architecture with residual and inverted residual block configurations is selected, and a set of data augmentation is applied to improve the dataset. Concerning feature selection, it adopts Binary Chimp Optimization and serial feature fusion regarding accuracy and speed. Experiments show that the proposed framework significantly outperforms conventional methods in the accurate classification of medicinal flora, and it suggests possible extensions for the identification of other plant species. This study provides evidence of the potential that deep learning models have in improving and automating identification and classification procedures for medicinal plants when integrated with botanical studies.

## 1 Introduction

Medicinal plants play a vital role in health care, acting as an integral resource both for traditional and modern medicines. According to the WHO, an estimated 35,000 to 70,000 plant species are used as medicine; this makes up about 14–28% of all 250,000 estimated plant species and 35–70% of the world's use of plants [1], [2]. Moreover, some 70% of people globally depend on plants to take care of their health primarily [3] because. These plants, whether wild or cultivated, are of therapeutic value because of their unique bioactive compounds that elicit physiological effects on the human body [4][5]. In fact, most locally grown medicinal plants form an integral component of food preparation and traditional medicine, hence socio-economically important [6]. Beyond health, medicinal plants are crucial to the conservation of their cultural, economic, and livelihood importance to people who depend on them. Accordingly, international guidelines and strategies have been designed for the conservation of medicinal plant biodiversity [7].

Accurate identification of medicinal plants is central to biodiversity conservation and in supporting traditional knowledge. Manual identification, however, becomes unreliable since some plant species are visually alike. Computer vision and machine learning open up possibilities for the creation of automated classification systems. The most popular application-based plant identification systems are LeafSnap and Pl@ntNet. However, all these methods still face issues in variability in plant appearance, such as leaf shape, color, and texture. Consequently, deep learning-based systems have been developed to improve the accuracy of plant classification [8], [9], [10].

Deep learning approaches, especially Convolutional Neural Networks (CNNs), have obtained tremendous success in image recognition tasks using large datasets with powerful computational models. The traditional machine learning techniques rely on handcrafted features derived from raw images, where CNNs learn hierarchical representations automatically—resulting in superior performances for plant species classification [11], [12].

Recent progress has steered the way towards hybrid models, which combine several deep learning architectures. Hybrid transfer learning models have been particularly effective in medicinal plant classification. The work of Ghosh et al. [13] demonstrated the effectiveness of hybrid transfer learning by using a combination of principal component analysis (PCA) and convolutional neural networks (CNNs). This method achieved a test accuracy of 95.25%, which outperformed the existing models in plant classification. Hybrid models can solve some of the important challenges in plant image datasets, such as high dimensionality and the heavy computation required to train deep networks from scratch. Hybrid models could improve the efficiency and accuracy of classification by adding PCA for dimension reduction and leveraging transfer learning from a pre-trained CNN. In this way, the approach drastically reduces computational costs yet still maintains high classification accuracy; hence, it becomes applicable in real-world applications to the classification of medicinal plants. There remains a big research gap to fill in the development of powerful automated systems used for the identification of medicinal plants. Although several works have been done using CNNs for image-based classifications, there is limited research that integrates multiple deep learning models, including hybrid models that fuse residual blocks, inverted residual blocks, and feature fusion. The current classification models, in many cases, assume large amounts of computational power and labeled data, sometimes not even available. The number of studies that relate to practical applications for these models, to support local communities and practitioners, is also few.

To fill this gap, our study proposes a hybrid deep-learning approach for medicinal plant classification. Our framework uses residual block and inverted residual block architectures to extract deep features from images. These features are further optimized by the Binary Chimp Optimization (BCO) algorithm to select the best features and perform dimensionality reduction. Besides, feature fusion is adopted to fuse the most discriminative features of both architectures so as to enhance classification performance.

Using the Grad-CAM (Gradient-weighted Class Activation Mapping) visualization technique, we highlight the image regions that contribute the most to the classification decision for a better understanding of the model's predictions. Our contributions brought by the proposed framework will be:

• We perform data augmentation over the selected dataset to generate high diversity in training images that help in model generalization.
• We design a hybrid architecture with residual blocks and inverted residual blocks for the efficient extraction of full contextual image features.
• We fuse these different sets of features according to their relevance by implementing the fusion strategy on both architectures: residual and inverted residual.
• We propose BCO in choosing the most discriminative features. Doing so, it further raises accuracy while lowering the complexity (computation cost).
• We apply machine learning classifiers on the optimized feature set for high-accuracy classification of medicinal plants.
• We apply Grad-CAM visualization to localize the most important regions of the plant images that are used in classification, so as to improve model interpretability.

The rest of this paper is organized as follows. Section 2 covers related work on medicinal plant classification and hybrid deep learning models. Section 3 expounds the system architecture, which includes data preprocessing, feature extraction, and classification methods. Section 4 presents experimental results, including model evaluation and comparison with other existing methods. Lastly, Section 5 summarizes the main findings and gives future research directions.

## 2 Related works

Research on plant leaf classification and disease detection has been greatly boosted in the past few years with the help of computer vision and machine learning. The current research landscape ranges from traditional image processing to state-of-the-art deep learning algorithms, each bringing its contribution to the ongoing quest for improved accuracy and robustness in the task of identifying plant species and detecting diseases. The following studies provide an overview of the development of feature extraction methods, classification models, and data processing techniques designed for plant leaf analysis—all in all, breadth, and adaptability of the current approaches in plant identification.

### 2.1 Traditional plant leaf classification methods

Computer vision has great promise to be applied in the study of plant leaves in identifying different plants and their diseases, where features play a vital role in classification. Wu et al. [14] introduced a method in which twelve morphological features were extracted from five basic geometrical features, and then applied PCA to lower the number of dimensions so that a probabilistic neural network has fewer inputs. They, along with their own Flavia dataset, achieved an average accuracy of 90.3%. Herdiyeni and Wahyuni [15] acquired a classification accuracy of 74.5% in the fusion of fuzzy local binary pattern, fuzzy color histogram, and a probabilistic neural network (PNN) classifier on the dataset of 2448 images (270 x 240 pixels) of leaves from medicinal plants taken from Indonesian forests. Ma et al. [16] proposed a method using a combination of Pyramid Histograms of Oriented Gradients (PHOG), Haar Wavelet Transform, and Top-hat transformation, reporting 90% accuracy for the classification of leaf images from the ImageCLEF 2012 dataset.

### 2.2 Deep learning models for plant leaf classification

Deep learning on the rise has automatically realized feature extraction from the traditional manual feature extraction. Grinblat et al. [17] obtained 92% recognition accuracy by classifying three different plant classes according to the vein pattern of leaves using a 3-layer CNN model. Paulson and Ravishankar et al. [18] identified 64 types of medicinal plants with a digital photo by three CNN deep learning models: VGG16, VGG19, and a custom CNN. Their accuracy rates were 95.7%, 97.8%, and 97.6%, respectively. Nasiri et al. [19] demonstrated that 99% accuracy can be achieved for the classification of grape leaves among six different cultivars by CNN models applied to visible range imaging recognition (400–700 nm). Hu et al. [20] developed a Multi-Scale Function (MSF) CNN model, which combined the multi-scale features with the CNNs in classifying leaves on plants. The MSF-CNNs are built with multiple learning branches having different learning scales. They conducted their experiment on MalayaKew (MK) and LeafSnap datasets, and their results showed the proposed MSF-CNN approach achieved state-of-the-art performances on plant leaf recognition.

### 2.3 Hybrid models and optimization techniques

Recent research has focused on hybrid models that integrate CNNs with optimization algorithms in order to improve classification performance. Ghosh et al. [13] proposed a Parallel Big Bang-Big Crunch (PB3C) CNN approach in which the PB3C algorithm was applied to optimize the CNN architecture for better classification accuracy and computational efficiency. Ghosh et al. [13] introduced the HPB3C-3PGA hybrid algorithm by combining PB3C with the 3-Parent Genetic Algorithm (3PGA) to speed up optimization for CNN architectures. Their method allows one to perform a robust exploration of the solution space while still holding on to global convergence, resulting in higher classification accuracy at reduced computational complexity. These hybrid approaches represent an increased interest in the combination of CNN models with metaheuristic optimization techniques for solving the computational burden associated with large-scale classification tasks.

### 2.4 Challenges and opportunities

Despite such development, challenges persist. A big challenge is how to handle intrinsic variability in the images of medicinal plants; differences in illumination, occlusions, and morphological variations within plants may cause loss in classification accuracy. Accordingly, data augmentation techniques are applied—such as rotation, flipping, and adding noise—to improve the robustness of the model to these challenges. Hybrid algorithms, such as HPB3C-3PGA, are promising for optimization problems. Due to the hybridization, through leverage in searching capabilities from multi-optimization algorithms, it fastens convergence towards the optimum solution and overcomes local minima. Our work takes further the same idea by introducing Binary Chimp Optimization onto the CNN architectures to raise the classification performance.

### 2.5 Summary of key contributions

The present study builds upon recent classification technique advances by using hybrid approaches for medicinal plant classification. The main contributions of this work include:

• **Combining Inverted Residual Blocks and Residual Blocks**: This architecture enhances the feature extraction phase, which increases the robustness and accuracy.
• **Application of Binary Chimp Optimization (BCO):** BCO algorithm selects the most relevant features of the feature map to guarantee that only crucial features are used for classification.
• **Serial-Based Feature Fusion:** This approach combines features of all network modules, which results in dramatically enriched feature representations and significantly better classification results. Consequently, hybrid models and optimization strategies utilized in the classification of medicinal plants hold significant relevance in integrating the latest outcomes from research, including those conducted by Ghosh et al. [13], among other studies. Our approach fills the gap in the existing literature by introducing an end-to-end classification system for medicinal plants, which foresees advancement with state-of-the-art architecture in CNNs and introduces optimization techniques, hence delivering a complete solution to problems classifying medicinal plants.

## 3 Proposed methodology

**Figure 1:**
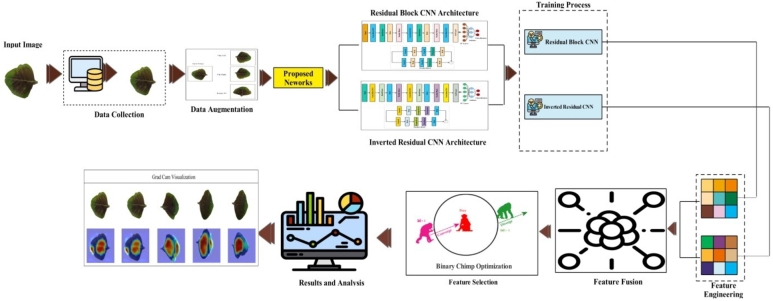
Proposed architecture of medicinal plant classification.

**Figure 2:**
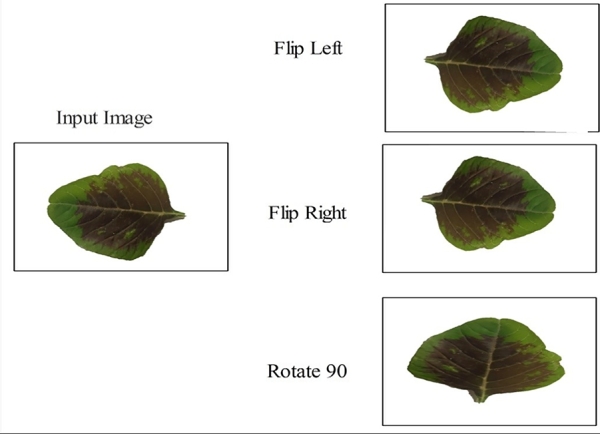
Data augmentation steps.

**Figure 3:**
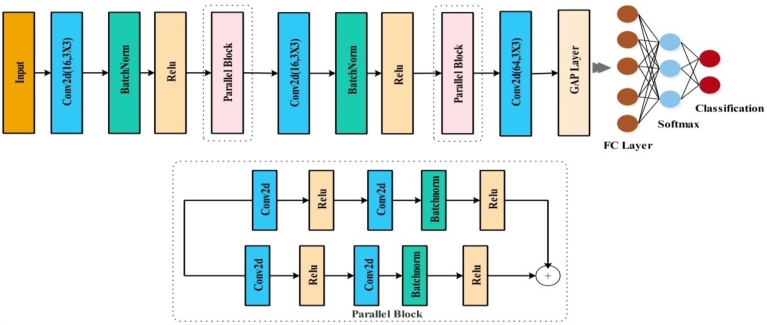
Proposed residual block architecture.

**Figure 4:**
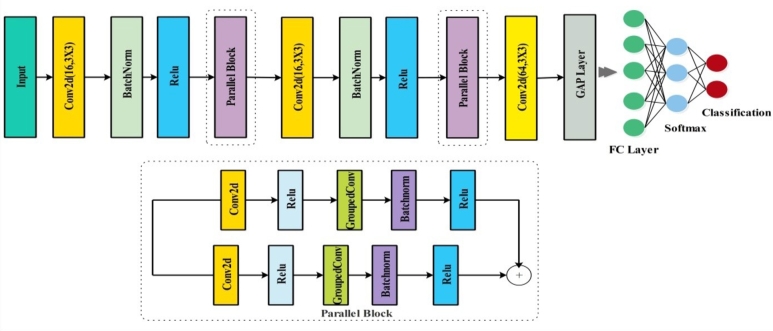
Proposed inverted residual block architecture.

Fig. 1 shows the images of medicinal plants that are used in the proposed scheme for classification. The proposed scheme is applied to conduct the first step of data augmentation on the chosen dataset of medicinal pictures Fig. 2. After the completion of the data augmentation process, the augmented dataset was fed into the two specially designed stream models based on the inverted residual block and self-attention mechanism Fig. 3. The features of the architecture based on self-attention mechanisms are extracted systematically, and those of the inverted residual block models are extracted; then, the sequential feature fusion method is applied to fuse the features of both customized models Fig. 4. The features are then fed into a machine learning system via the Binary Chimp optimization feature selection method, which allows for choosing highly appropriate features. Grad-Cam visualization is further used to localize healthy and diseased regions in the sample images. The detailed steps of the proposed approach are described below.

### 3.1 Dataset collection and augmentation

A publicly available dataset of medicinal images classifications is used in the experimental technique of this study https://www.kaggle.com/datasets/sharvan123/medicinal-plant -30 classes. Therefore, these datasets are not balanced. To solve this issue, it is recommended to apply data augmentation techniques. To validate the main findings, this study uses medicinal datasets. The collection contains 1000 images with an average pixel size of 500x500. In addition, the training and testing data sets are divided in half. Consequently, these data sets cannot be used to train the deep learning model. Thus, through Fig. 2, we can get data augmentation by Flip Left, Flip Right, and Rotate 90. Repeats the above steps in every data set until it gets enough photos for training.

### 3.2 Proposed residual block architecture

The Residual Block forms one of the significant building blocks of MobileNetV2, a very effective neural network architecture for mobile and embedded vision applications. Since it reduces computational costs and raises performance, the block would be proper for devices of low computing power. The proposed framework was developed by using a two-parallel-block-based residual block architecture Fig. 1. The proposed CNN architecture is based on an inverted residual block structure and has an input size of 224 × 224 × 3. After the input, the convolutional layer and batch normalization are applied by using a 3 × 3 kernel size, 16 depths, and a 2 × 2 stride. First, we have two parallel convolution layers, batch normalization, and RELU activation in the first parallel block. The first parallel block has a 3 × 3 kernel size, 16 depth sizes, and a 1 × 1 stride. The second inverted residual block includes two parallel convolutional layers, one batch normalization layer with a depth of 32, and makes use of ReLU activation. The kernel size is 3 × 3 with a stride of 1 × 1. Moreover, extra connections are created between the grouped convolution layer and the fully connected, softmax, global average pooling, and classification layers. This model has 258k parameters, or 22 of the 45 layers are convolutional layers. The deep properties of the global average pool layer are deleted, and the proposed model is trained by both datasets. N × 1024 is the dimension of the extracted features. The architecture of the proposed residual-based CNN is shown in Fig. 3.

### 3.3 Proposed inverted residual block architecture

For this new framework, we developed a three parallel blocks-based CNN residual block architecture. Our proposed CNN architecture is constructed based on inverted residual block architecture and the input size is 224 × 224 × 3. After the input, batch normalization and the convolutional layer are used with 2 × 2 stride, 16 depths and the kernel size of 3 × 3. At first, the initial parallel block consists of two parallel convolutional layers, batch normalization, and a RELU activation function, which has a kernel size of 3 × 3, a depth of 16, and a stride of 1 × 1. The following structure of the inverted residual block contains a 3 × 3 kernel with a stride of 1 × 1, combined with two parallel convolutional layers, one batch normalization with a depth of 32, and RELU activation. Two convolutional layers, two levels of RELU activation, and one batch normalization layer are coupled to the third parallel block, which starts with RELU activation. The same padding, a 3 × 3 kernel with a 1 × 1 stride, and a 64-depth size are related to these layers with each other. The architecture of the proposed Inverted residual-based CNN is depicted in Fig. 4.

### 3.4 Serial based feature fusion

Serial-based feature fusion is a technique in computer vision and deep learning. It integrates features from multiple sources or layers in a step-by-step manner to improve the representation capacity of the model. This fusion technique allows the network to discover complex patterns and relationships, which can be learned from the combined data by combining different stages or layers of features. Such in applications like object detection, picture segmentation, and video analysis where one seeks gathering diversified and complementary information to aid in making good predictions, serial-based feature fusion becomes very helpful. Mathematically expressed as:

Now let's assume, momentarily, the presence of two feature vectors, a and b, of size k×N, where *k* is the number of features and *N* is the number of observations.

(1) $$S_{\left(v\right)}^{fuse}={\left[\frac{k}{N}\right]}_{N\times k1+N\times k2}$$

The best DA features are selected in the fusion process based on entropy. The final vectors after fusion are N× 1024 and N× 1024. Finally, fused features are inputted into classifiers through neural networks, which use them to find out how many observations have been classified into each category.

### 3.5 Feature optimization (BCO)

In 2020, Khishe and Mosavi introduced a new swarm intelligence-based optimization algorithm called ChOA. The inspiration behind ChOA is the sexual instincts and cognitive capabilities of chimpanzees, which, unlike other social predators, make them unique because of their group hunting behavior. Due to its simplicity, fast convergence, ability to escape local optima, and low computational cost, this algorithm has been applied to a wide range of complex optimization problems. Chimpanzee hunting behavior can be characterized by two distinct phases, which are: exploration and exploitation. The exploitation phase involves the actual engagement with the prey that offers feasible local search opportunities identified during the exploration phase. However, exploration involves movements, creating barriers, and chasing prey to uncover a larger area within the global search environment. The four classes of chimpanzees that participate in these hunting steps are the attacker, the barrier, the chaser, and the driving groups. Each chimpanzee in the population represents a candidate solution in the given search space; more precisely, the attacker, barrier, chaser, and driver chimpanzees, in that order, stand for the optimum (leader), the second optimum, the third optimum, and the fourth optimum solutions, respectively. The best four chimpanzees named: the attacker ($a_{\mathrm{attacker}}$), the barrier ($a_{\mathrm{barrier}}$), the chaser ($a_{\mathrm{chaser}}$) and the driver ($a_{\mathrm{driver}}$) are used to guide the movement of the remaining chimpanzees ($a_{\mathrm{chimp}}$) at the start and end of each iteration. This process is achieved through the following formulas.

(2) $$x1^{\left(t+1\right)}=a_{{\mathrm{attacker}}^{\left(t\right)}}-A1.(D_{\mathrm{attacker}}),D_{\mathrm{attacker}}=C1.x_{\mathrm{attacker}}-m.x_{{\mathrm{chimp}}^{\left(t\right)}}$$

(3) $$x2^{\left(t+1\right)}=a_{{\mathrm{barrier}}^{\left(t\right)}}-A2.(D_{\mathrm{barrier}}),D_{\mathrm{barrier}}=C2.x_{\mathrm{barrier}}-m.x_{{\mathrm{chimp}}^{\left(t\right)}}$$

(4) $$x3^{\left(t+1\right)}=a_{{\mathrm{chaser}}^{\left(t\right)}}-A3.(D_{\mathrm{chaser}}),D_{\mathrm{chaser}}=C3.x_{\mathrm{chaser}}-m.x_{{\mathrm{chimp}}^{\left(t\right)}}$$

(5) $$x4^{\left(t+1\right)}=a_{{\mathrm{driver}}^{\left(t\right)}}-A4.(D_{\mathrm{driver}}),D_{\mathrm{driver}}=C4.x_{\mathrm{driver}}-m.x_{{\mathrm{chimp}}^{\left(t\right)}}$$

(6) $$x_{{\mathrm{chimp}}^{\left(t+1\right)}}=\frac{a1+a2+a3+a4}{4}$$

where $x_{chimp}$ shows the location of each solution in iteration *t* and *t* is the number of the current iteration.

**Algorithm 1:**
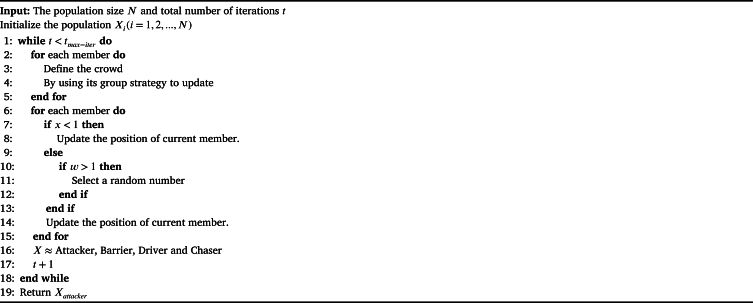
Pseudo-code of ChoA algorithm.

## 4 Results and analysis

This section presents the experimental results of the proposed scheme in detail. Experiments are conducted using medicinal flora-related datasets; therefore, this section has a detailed discussion of the results obtained using each dataset. Each dataset is divided into two halves. This division means that one-half of the images is used for testing and the other half for training the proposed model. In all experiments, 10-fold cross-validation was applied—a procedure commonly adopted to achieve a good trade-off between computational cost and variance, the latter being related to the generalizability of the performance estimate. An overall × 1024 feature dimensions were used in the experiment; a performance score of 10 was obtained by setting k to this particular value, while using a smaller value of k was found to be ineffective. For each exploration, ten iterations of k-fold cross-validation were carried out. The suggested models are trained by the following parameters: optimizer, mini-batch size, learning rate and epochs with values of SGDM 16, 0.0001 and 10 respectively. All experiments are evaluated based on time, f1-score, precision, recall, accuracy, error, false negative rate. All testing was conducted using the MSI Leopard series motherboard housing an Intel Core i7 processor, 16 GB of RAM, a 512 GB SSD with integrated 1 TB HDD and a 4 GB graphics card from NVIDIA RTX.

### 4.1 Proposed residual block architecture results

**Figure 5:**
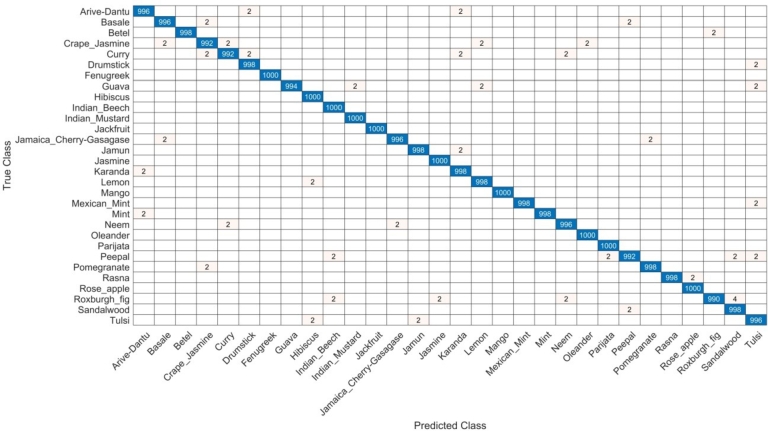
Confusion matrix of WNN classifier of residual block architecture.

The first part discusses the results obtained from the medicinal plants dataset. The components are from the proposed architecture on a residual block derived from CNN. The performances of the Residual Block CNN model are summarized in Table 1. In this table, the WNN classifier performed best with an accuracy of 99.7%. Moreover, the precision, recall, and F1-score were 99.6%, 99.6%, and 99.6%, respectively, with a computational time of 669.05 seconds. Reaching an accuracy of 85.8% for NNN, the other classifier had precision rate, sensitivity, and F1-score of 85.7 and 85.8 respectively. The computational times for each classifier were 543.94(s), 637.68(s), 583.3(s), and 596.99(s). Fig. 5 shows the confusion matrix for the WNN classifier.

**Table 1:** Proposed residual block architecture results.

NNN	85.7	85.8	85.7	85.8	543.94(s)
MNN	98.2	98.1	98.2	98.2	637.68(s)
**WNN**	**99.6**	**99.6**	**99.6**	**99.7**	**669.05(s)**
BNN	84.2	84.1	84.1	84.4	583.3(s)
TNN	82.3	82.4	82.3	82.4	596.99(s)

### 4.2 Proposed inverted residual block architecture results

**Figure 6:**
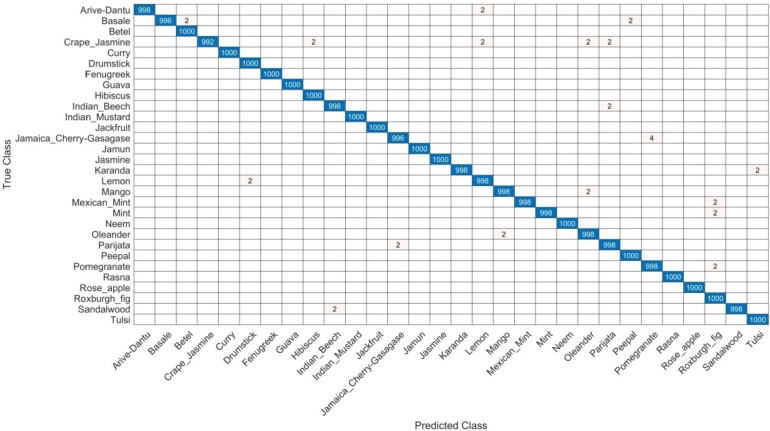
WNN classifier of proposed inverted residual block architecture results.

The next section presents the results obtained from the medicinal plants data set. The components are outputs of the proposed architecture using CNN-based inverted residual blocks. The results of the CNN-based inverted residual block design are presented in Table 2. In this table, the WNN classifier performed better and achieved an accuracy of 99.9%. With the computation time of 343.74(s), accuracy rate, recall rate, and F1-score are 99.8%, 99.9%, and 99.8% respectively. The rest of the classifier reached an accuracy of 97.6% for the NNN, with the precision rate, sensitivity, and F1-score of 97.5, 97.6, and 97.5 respectively. The computational time for each classifier was 597.91(s), 312.98(s), 638.96(s), and 647.99(s). The confusion matrix for the WNN classifier is illustrated in Fig. 6.

**Table 2:** Proposed inverted residual block architecture results.

NNN	97.5	97.6	97.5	97.6	597.91(s)
MNN	99.7	99.8	99.7	99.8	312.98(s)
**WNN**	**99.8**	**99.9**	**99.8**	**99.9**	**343.74(s)**
BNN	96.4	96.5	96.5	96.9	638.96(s)
TNN	95.2	95.1	95.1	95.3	647.99(s)

### 4.3 Serial based feature fusion results

**Figure 7:**
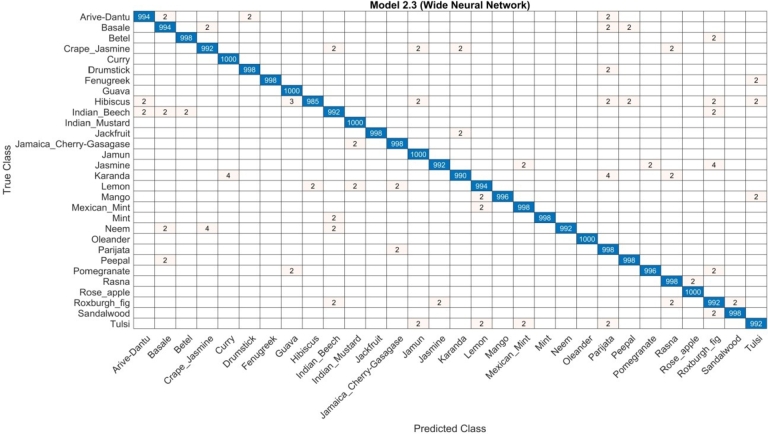
Serial based feature fusion results.

The experimental results of the medicinal plants dataset are shown in third part. The features are derived from the introduced serial-based feature fusion. The experimental results of the feature fusion are shown in Table 1. The WNN classifier in Table 1 performed well and the accuracy rate was 99 6%. The accuracy rate, the recall rate and the F1 score were 99. 4%, 99. 5% and 99. 6%, respectively, when the computation time was 1586.5(s). For the rest classifier, the accuracy obtained for the NNN is 85.3%, MNN is 98.1%, and BNN and TNN achieved an accuracy of 84. 7% and 81. 2%. The computational time of each classifier had a result of 1328.2(s), 1534.4(s), 1233(s) and 1274.7(s) (Table 3). The confusion matrix of the WNN classifier is shown in Fig. 7.

**Table 3:** Serial based feature fusion results.

NNN	85.3	85.2	85.3	85.3	1328.2(s)
MNN	97.9	98.0	98.1	98.1	1534.4(s)
**WNN**	**99.5**	**99.4**	**99.6**	**99.6**	**1586.5(s)**
BNN	84.5	84.6	84.7	84.7	1233(s)
TNN	81.1	81.0	81.2	81.2	1274.7(s)

### 4.4 Feature optimization results

**Figure 8:**
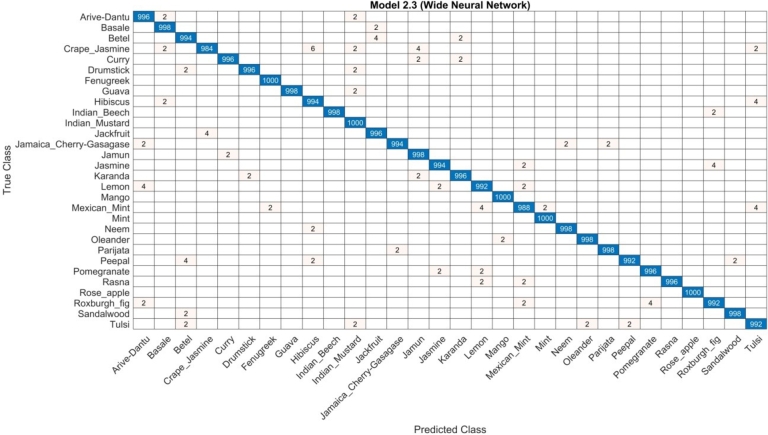
Feature optimization results.

The last part shows the results of the medicinal plant dataset. The suggested feature optimization outcomes are the source of the components. Table 4 lists the conclusions of the feature fusion analysis. In the table, the WNN classifier was optimal with an accuracy of 99.6%. The accuracy rate, the recall rate and the F1 score were 99. 4%, 99. 5% and 99. 6%, respectively, with a computation time of 1397.2 (s). The precision of the rest of the classifier was 80.0% for NNN, 93.1% for MNN and 79.6% and 77.0% for BNN and TNN, respectively. The computational times for each classifier were 1048.7 (s), 1190.5 (s), 996.73 (s) and 1020.6 (s), respectively. Fig. 8 shows the confusion matrix for the WNN classifier.

**Table 4:** Feature optimization results.

NNN	79.9	79.8	79.8	80.0	1048.7(s)
MNN	93.0	93.1	93.1	93.1	1190.5(s)
**WNN**	**99.5**	**99.4**	**99.6**	**99.6**	**1397.2(s)**
BNN	79.5	79.6	79.6	79.6	996.73(s)
TNN	77.0	77.0	77.0	77.0	1020.6(s)

### 4.5 Grad cam visualization

**Figure 9:**
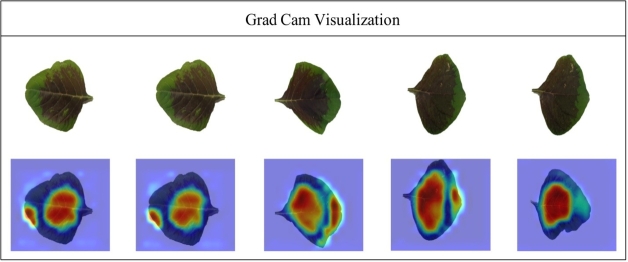
Grad cam visualization.

Finally, the proposed deep learning model is presented. The gradient visualization of the proposed deep learning model is obtained by using Grad-CAM visualization, as shown in Fig. 9. From the Grad-CAM, it can be observed that there is an application of a heatmap to the relevant parts in this visualized representation. The region most relevant to the effectiveness of the model is represented by the color brown. This picture verifies that the proposed model correctly predicts the classes.

## 5 Conclusion

The research highlights the crucial role of medicinal plants in the health sector and the need for effective classification methodologies to protect and enhance them. Using deep learning methods, especially transfer learning, this study presents an example of remarkable improvements in the levels of precision and precision of medicinal plant classification. The adoption of advanced convolutional neural networks (CNNs), together with new architectural designs such as residual and inverted residual blocks, has further improved the efficacy in classification. Results like these show that the integration of machine learning techniques with botanical studies will be able to lift the conservation and application of medicinal flora, and also contribute positively to health care and biodiversity conservation.

Future research efforts will focus on the integration of multimodal data by combining leaf imagery with other botanical features, such as chemical makeup and genetic data, to improve the accuracy of classification and allow for species discrimination. This will also include developing more robust models that can be adapted to different environmental conditions, including changes in lighting, background, and plant maturity, to improve the generalization of the medicinal plant classification systems. Further expansion of our data set to include underrepresented plant species and the conducting of cross-regional studies will contribute to the creation of a more holistic model. Lastly, we will apply these advanced classification techniques in mobile applications or real-time field devices to make them usable and pragmatic tools for researchers, practitioners, and conservationists in the identification and preservation of medicinal plants in their natural environment.

## CRediT authorship contribution statement

**Hicham Bouakkaz:** Writing – original draft, Resources, Investigation, Formal analysis, Data curation. **Mustapha Bouakkaz:** Writing – original draft, Supervision, Methodology. **Chaker Abdelaziz Kerrache:** Writing – review & editing, Methodology, Investigation. **Sahraoui Dhelim:** Writing – review & editing, Validation, Formal analysis.

## Declaration of Competing Interest

The authors declare that they have no known competing financial interests or personal relationships that could have appeared to influence the work reported in this paper.

## Data Availability

No new data was generated for the research described in the article.
